# Efficient *in vivo* antitumor effect of an immunotoxin based on ribotoxin α-sarcin in *nude* mice bearing human colorectal cancer xenografts

**DOI:** 10.1186/s40064-015-0943-5

**Published:** 2015-04-08

**Authors:** Jaime Tomé-Amat, Miriam Olombrada, Javier Ruiz-de-la-Herrán, Eduardo Pérez-Gómez, Clara Andradas, Cristina Sánchez, Leopoldo Martínez, Álvaro Martínez-del-Pozo, José G Gavilanes, Javier Lacadena

**Affiliations:** Departamento de Bioquímica y Biología Molecular I, Facultad de Ciencias Químicas, Universidad Complutense de Madrid, Avenida Complutense s/n, Madrid, 28040 Spain; Present address: Department of Microbiology, Global Health and Emerging Pathogens Institute, Icahn School of Medicine at Mount Sinai, New York, NY USA; Instituto de Investigación Hospital 12 de Octubre, Madrid, 28041 Spain; Hospital Universitario La Paz, Madrid, Spain

**Keywords:** Immunotoxin, *in vivo* antitumor effectiveness, Colorectal cancer, GPA33, Ribotoxin α-sarcin

## Abstract

Tagging of RNases, such as the ribotoxin α-sarcin, with the variable domains of antibodies directed to surface antigens that are selectively expressed on tumor cells endows cellular specificity to their cytotoxic action. A recombinant single-chain immunotoxin based on the ribotoxin α-sarcin (IMTXA33αS), produced in the generally regarded as safe (GRAS) yeast *Pichia pastoris*, has been recently described as a promising candidate for the treatment of colorectal cancer cells expressing the glycoprotein A33 (GPA33) antigen, due to its high specific and effective cytotoxic effect on *in vitro* assays against targeted cells. Here we report the *in vivo* antitumor effectiveness of this immunotoxin on *nude* mice bearing GPA33-positive human colon cancer xenografts. Two sets of independent assays were performed, including three experimental groups: control (PBS) and treatment with two different doses of immunotoxin (50 or 100 μg/ injection) (n = 8). Intraperitoneal administration of IMTXA33αS resulted in significant dose-dependent tumor growth inhibition. In addition, the remaining tumors excised from immunotoxin-treated mice showed absence of the GPA33 antigen and a clear inhibition of angiogenesis and proliferative capacity. No signs of immunotoxin-induced pathological changes were observed from specimens tissues. Overall these results show efficient and selective cytotoxic action on tumor xenografts, combined with the lack of severe side effects, suggesting that IMTXA33αS is a potential therapeutic agent against colorectal cancer.

## Introduction

Colon cancer is among the most deadly ones with a significant worldwide incidence. Its treatment by immunotherapy is becoming relatively successful with three monoclonal antibodies already approved for clinical use (Eng 2010; Tol and Punt 2010; Sliwkowski and Mellman 2013). However, its late diagnosis and metastatic progression makes the development of more efficient drugs necessary. In this scenario, immunotoxins are highly specific therapeutic agents that hold promise as antitumoral agents. They combine the specificity of an antibody fragment with the potency of a toxin, causing death of the target cells (Pastan et al. 2007; Dougan and Dranoff 2009).

Immunotherapeutic approaches using antibodies have been widely explored against a variety of tumors but an effective treatment of solid tumors still remains as a problem because therapeutic antibodies must diffuse into tumors through a disordered vasculature and against a hydrostatic pressure gradient (Jain 2001; Dienstmann et al. 2012). Immunotoxins design has greatly evolved focused on the targeting domain, mainly towards a better penetration in solid tumors or an increase of immunotoxins stability or functionality (Onda et al. 2011; Gehlsen et al. 2012). Because low–molecular weight antibody fragments have been shown to have better tumor diffusion properties, single-chain variable fragments (scFv) have been favored to deliver protein-based toxins to cancer cells (Madhumati and Verma 2012; Sapra and Shor 2013).

GPA33 is an extensively studied membrane antigen (Heath et al. 1997) overexpressed in 95% of primary or metastatic colorectal cancers and absent in most of any other tissue, tumoral or not. Due to its features, GPA33 represents an ideal target for immunotoxins aimed against colon cancer cells (Scott et al. 2005; Ackerman et al. 2008). In fact, the three mAbs approved for colon cancer immunotherapy, bevacizumab and cetuximab or panitumumab, are not completely specific for target colon cancer, as they recognize the vascular endothelial growth factor (VEGF) or the epidermal growth factor receptor (EGFR), respectively (Sliwkowski and Mellman 2013).

Different A33 humanized monoclonal antibody (huA33) based constructions against GPA33 antigen have been described, including several clinical assays with radioimmunoconjugates using the whole antibody molecule (Welt et al. 2003; Scott et al. 2005; Almqvist et al. 2006), recombinant scFv designs for Antibody-Directed-Enzyme-Prodrug-Therapy (ADEPT) (Coelho et al. 2007; Panjideh et al. 2008) or, more recently, preclinical assays with immuno-targeted gold-iron oxide hybrid nanoparticles for therapeutic strategies (Kirui et al. 2013).

Regarding the toxic moiety, human or fungal ribonucleases (RNases) have become a new alternative for their use in this domain as opposed to the commonly used ricin and *Diphtheria* or *Pseudomonas* toxins (Ardelt 2013), which in many cases show immunogenic reactivity or undesirable side effects (Frankel et al. 2000; Schindler et al. 2001; Onda et al. 2011). In this sense, ribotoxins are cytotoxic fungal extracellular RNases with α-sarcin as its most outstanding member (Lacadena et al. 2007; Olombrada et al. 2014a). They behave as potent inhibitors of protein biosynthesis due to its highly specific ribonucleolytic activity, which cleaves a single phosphodiester bond of the larger molecule of rRNA located at a universally conserved site, known as the sarcin/ricin loop (SRL), leading to cell death by apoptosis (Schindler and Davies 1977; Lacadena et al. 1999; Olmo et al. 2001; García-Ortega et al. 2010; Olombrada et al. 2014b)

Ribotoxins have several advantages for their use as immunotoxins toxic moiety. Namely, their small size, high thermostability, poor immunogenicity, resistance to proteases and highly efficient ability to inactivate ribosomes (Rathore and Batra 1996; Goyal and Batra 2000; Lacadena et al. 2007; Carreras-Sangrà et al. 2008). Thus, different ribotoxins have been used before as components of immunotoxins, and assayed *in vitro* (Orlandi et al. 1988; Wawrzynczak et al. 1991; Better et al. 1992; Rathore et al. 1997; Goyal and Batra 2000).

Recently, we have reported the first ribotoxin-based-scFv recombinant immunotoxin directed against human colorectal cancer cells by the fusion of humanized scFvA33 and α-sarcin (IMTXA33αS) (Carreras-Sangrà et al. 2012). Incubation with nanomolar concentrations resulted in a very high specific and effective cytotoxicity against targeted cells in *in vitro* assays.

The results herein presented, in terms of its *in vivo* specific antitumor effect using *nude* mice harbouring colon cancer xenografts, reveal a great antitumor effectiveness with a strong inhibition of tumor growth, angiogenesis and proliferative properties.

## Results

### IMTXA33αS inhibits protein synthesis in vitro

IMTXA33αS production and structural and *in vitro* functional characterization have been previously reported (Carreras-Sangrà et al. 2012). IMTXA33αS shows a highly specific and effective cytotoxicity against colon cancer target cells (SW1222 or LIM1215) overexpressing the GPA33 antigen, with IC_50_ (protein concentration inhibiting 50% of protein synthesis) values of 30 and 70 nM, respectively. Unlike, free wild type α-sarcin exhibits no difference between negative and positive antigen cells, with IC_50_ values of ≥1 μM (Carreras-Sangrà et al. 2012). Moreover, when we analyzed the cytotoxicity of the scFvA33 alone against GPA33-positive SW1222 cells cultures IC_50_ value was too high to be measurable (Table 1). Thus, as expected, the scFvA33 target was not able to induce death of the targeted cells, unless fused to α-sarcin as part of the immunotoxin.

**Table 1:** ***In vitro*** **cytotoxicity of IMTXA33**α**S, wild type** α**-sarcin and scFvA33**

	**GPA33 (+)**		**GPA33 (−)**	
	**SW1222**	**LIM1215**	**HT29**	**A431**
IMTXA33αS	0.03*	0.07	> > 1.00*	> > 1.00*
α-Sarcin	0.8*	> 1.00	> > 1.00*	1.00*
scFvA33	NM	NM	NM	NM

*, (Carreras-Sangrà et al. 2012); NM, too high to be measurable.

IC_50_ (protein concentration inhibiting 50% of protein biosynthesis) (μM) obtained for the cell lines assayed, after 72 h of incubation with the different proteins.

### IMTXA33αS reduces tumor growth in vivo

**Figure 1:**
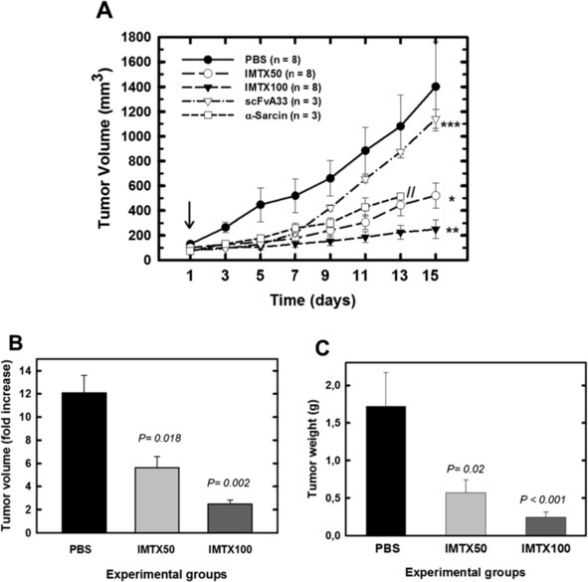
**Immunotoxin inhibits colorectal tumor growth** ***in vivo*** **. A)** Time course of the tumor volume progression of SW1222-derived xenografts non-treated (PBS) or treated with 50 or 100 μg IMTXA33αS (IMTX50 or IMTX100, respectively), 40 μg wild type α-sarcin or 61 μg scFvA33, per injection. α-Sarcin and scFvA33 doses were equivalent to the highest dose of immunotoxin used (IMTX100), 2.2 nanomoles in each case. The arrow indicates the beginning of treatment Doses were given every 48 hours. *, p < 0.05; **, p < 0.01; ***, p > 0.05 *vs* vehicle treated tumors. //, indicates suspension of treatment. **B)** Growth and **C)** weight measurements of excised tumors of non-treated (PBS) or after *in vivo* treatment with IMTXA33αS.

To study the *in vivo* effect of the immunotoxin, the development of solid tumors was induced in *nude* mice by inoculation of SW1222 cells, as GPA33-positive cell model, in the rear right flank. SW1222-xenografts started to appear approximately seven days after cell injection with a significant and rapidly heterogeneous growth as previously described (Barendswaard et al. 2001). Mice with palpable tumors of 50–100 mm^3^ of volume were challenged with immunotoxin treatment as described in Materials and Methods. Wild type α-sarcin and scFvA33 were also included as controls in the *in vivo* assay (Figure 1A). As expected, scFvA33 administration did not affect tumor growth except from a slight delay, which was not significant from a statistical point of view. By contrast, treatment with wild type α-sarcin produced a significant inhibition of tumor growth, but treatment had to be suspended because of toxicity in mice, which showed skin disorders, weight loss and decreased mobility.

We then analyzed the effect of two doses of immunotoxin (IMTX50 and IMTX100 experimental groups) on tumor progression compared with the vehicle group (PBS) (Figure 1A). Treatment with the immunotoxin strongly slowed down tumor growth in a dose-dependent manner. At the end of the treatment, tumor volume in animals challenged with IMTX50 and IMTX100 was reduced up to 3 to 6 times, respectively, as compared to vehicle-treated animals (Figure 1B). Similar results were obtained when tumor weight was considered (Figure 1C). Tumors were analyzed by H&E staining showing the presence of healthy tumor cells and well-vascularized tissues with no apparent cell damage in non-treated mice, as opposed to the cell damage and necrotic features observed in the immunotoxin-treated tumor cells (data not shown).

### IMTXA33αS reduces GPA33 antigen expression, angiogenesis and cancer cell proliferation in vivo

**Figure 2:**
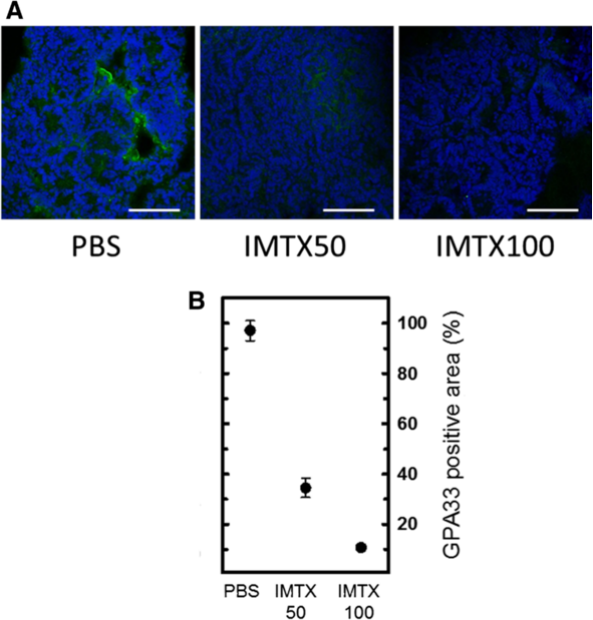
**IMTXA33** **α** **S-treated tumors show lack of GPA33 antigen** ***in vivo*** **. A)** GPA33-positive cells (green). Scale bars: 50 μm. **B)** Quantification of GPA33-positive cells. Statistical analysis of IMTX50 or IMTX100-treated tumors *vs* vehicle-treated tumors rendered p <0.001.

The analysis of the tumors revealed that the percentage of GPA33-positive cells in the tumors showed a large decrease after treatment with the immunotoxin, due to death of target cells (Figure 2), with less than 10% of positive area for the highest dose. Thus, most of the remaining cells from these tumors lost the GPA33 antigen and, presumably, the tumor aggressiveness.

**Figure 3:**
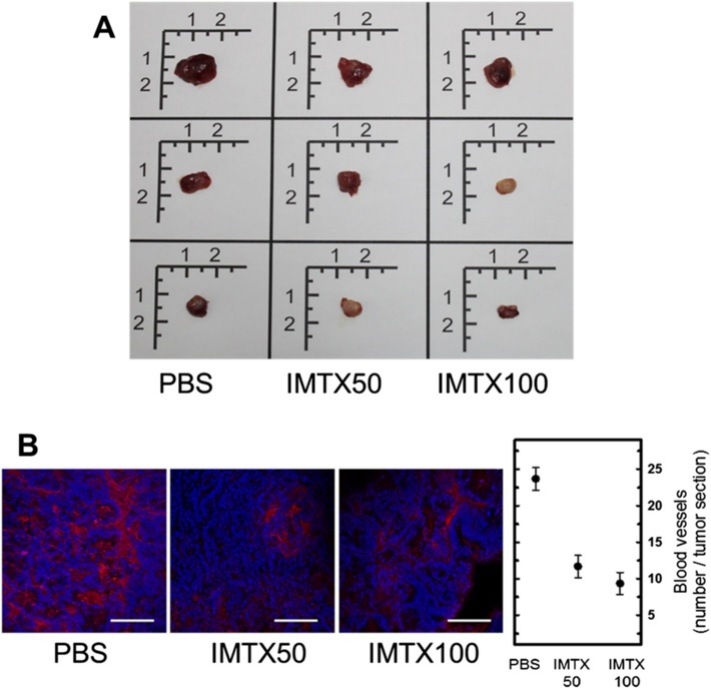
**IMTXA33** **α** **S impaired angiogenesis on** ***in vivo*** **treated tumors. A)** Representative images of the excised remaining tumors of non-treated (PBS) or treated (IMTX50, IMTX100) experimental groups. Scale is in centimeters. Each column shows three examples from each group. **B)** CD31-positive cells (red). Scale bars: 60 μm. Quantification of the number of blood vessels is shown in the corresponding graph. Statistical analysis of IMTX50 or IMTX100-treated tumors *vs* vehicle-treated tumors rendered p <0001.

Interestingly, when the animals were sacrificed and the remaining tumors were excised significant differences in their morphologic features were evident. Tumors from the non-treated group were solid and well vascularized. On the other hand some of the IMTX100-treated mice tumors were more whitish, squishy and cyst-like (Figure 3A). This colour differences were suggestive of effects on the angiogenic process. In fact, tumor vascularization was impaired by the immunotoxin, as the number of blood vessels was smaller, as determined by CD31 staining (Figure 3B), confirming what was observed in the macroscopic analysis.

**Figure 4:**
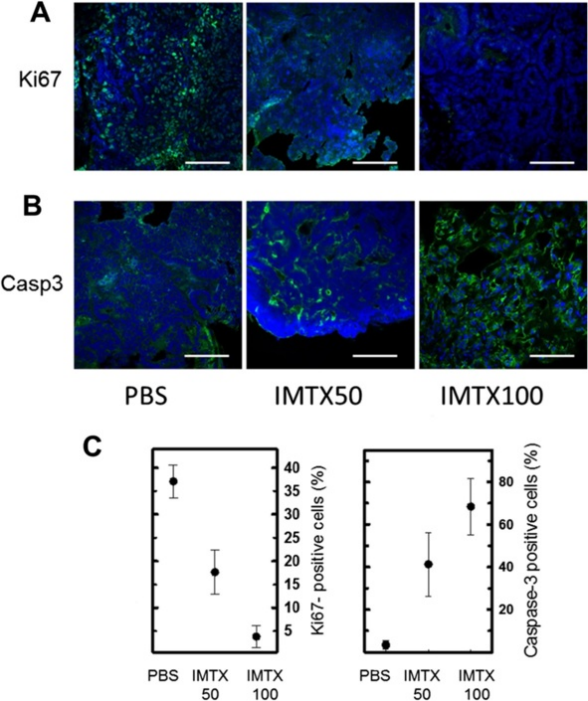
**IMTXA33**α**S-treated tumors show inhibition of cancer cell proliferation and induction of cancer cell apoptosis** ***in vivo*** **. A)** Ki67-positive cells (green) and **B)** active caspase-3-positive cells (green) in tumors. Scale bars: A, 60 μm; B, 60, 30, 40 μm (from left to right). **C)** Quantification of Ki67-positive cells **(A)** and active-caspase-3-positive cells **(B)** in the tumors are shown in the corresponding graphs. Statistical analysis of IMTX50 or IMTX100-treated tumors *vs* vehicle-treated tumors rendered p < 0.001 **(A)** and p < 0.05 **(B)**.

The proliferative potential of cancer cells present in the tumors was also analyzed. A significant reduction of this potential was again observed in immunotoxin-treated tumors, as indicated by the dose dependent decrease in the number of Ki67-positive cells (Figure 4A and C). Moreover, immunotoxin administration increased the number of active caspase 3-positive cells, indicating tumor cells death by apoptosis (Figure 4B and C).

### IMTXA33αS does not produce adverse side effects

**Figure 5:**
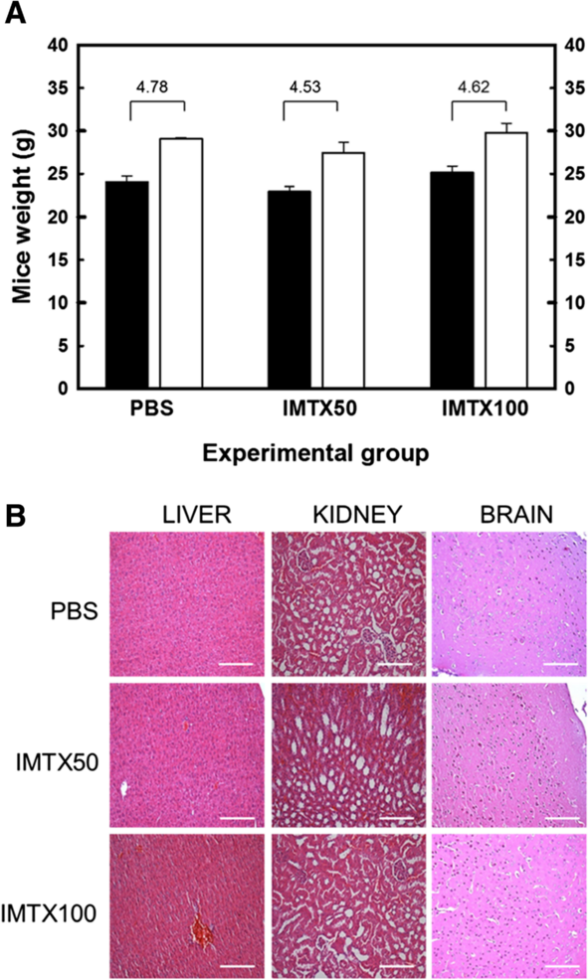
**IMTXA33**α**S-treated mice show no major toxicity effects** ***in vivo*** **. A)** Mice weight measurement before and after treatment. Black bars (before treatment), white bars (after treatment). Increase in weight (g) is indicated for each experimental group. No significant statistical differences were observed between the different experimental groups. **B)** Hematoxylin & eosin staining of representative tissue sections from non-treated (PBS) or treated- (IMTX50 or IMTX100) mice. Scale bars: 120 μm (liver), 40 μm (kidney) and 100 μm (brain). No significant differences in histological tissue analysis of the selected organs were observed.

Growth of the mice was not affected by treatment with IMTXA33αS in any of the doses assayed, considering their weight (Figure 5A) and external appearance. In this sense, regardless of the group to which they belonged, weight was increased in about 20% at the end of the treatment.

None of the mice showed histomorphologic changes in a macroscopic analysis resulting from immunotoxin treatment. Moreover no significant changes were observed in the histological analysis by H&E staining in any of the organs analyzed, regardless of the experimental group they belonged (Figure 5B).

## Discussion

ScFv-immunotoxins have been lately used for experimental therapeutic approaches due to its more favourable penetration and diffusion features in solid tumor tissues. Within this idea, we have recently reported the high specificity and effectiveness on *in vitro* assays of two recombinant scFv-immunoconjugates, based on the fungal RNases α-sarcin and RNase T1, against colon cancer targeted cells (Carreras-Sangrà et al. 2012; Tomé-Amat et al. 2012).

In this work we move one step further and demonstrate the potential of IMTXA33αS for *in vivo* applications, using SW1222-induced xenografts, as a model of GPA33-positive colon cancer. The results have shown a clear inhibition effect on tumor growth accompanied by a significant decrease in the proliferative and angiogenic capacities of the treated tumors, as determined by Ki67 and CD31 labeling. The inhibition of protein biosynthesis in immunotoxin-treated tumors, due to α-sarcin specific RNase activity, led to apoptosis causing cell death, as determined by active-caspase 3 labeling. This mechanism is a well-known feature of the ribotoxic action of this toxin (Olmo et al. 2001; Lacadena et al. 2007).

Furthermore, treatment with the highest dose of immunotoxin resulted in a residual tumor mass, lacking the GPA33 antigen and with a cyst-like appearance. GPA33 belongs to the immunoglobulin superfamily with homology to tight junction-associated proteins. It’s closest homologous include the junction adhesion molecules (JAM) or the CEA-related cell adhesion molecules, among others (Ackerman et al. 2008), which are involved in tumor proliferation (Goetsch et al. 2013) or tumor angiogenesis (Kuespert et al. 2006), respectively. Thus, the absence of the GPA33 antigen after treatment with IMTXA33αS would be consistent with the observed anti-angiogenic effect and the decrease in the proliferative potential of the residual tumor mass. These results would suggest a correlation between GPA33-expression level and aggressiveness or the tumor, as previously described for JAM in relation to poor prognosis of breast cancer (McSherry et al. 2009).

Moreover, along with the high antitumor effectiveness of IMTXA33αS, adverse side effects were not observed at the histological level or in the development of the treated mice. Thus the scFvA33 target domain directs α-sarcin to the target cells avoiding the nonspecific toxicity shown in the *in vivo* assay with free wild type α-sarcin (Figure 1). Similar effects have been previously described for free wild type α-sarcin and other ribotoxins, like mitogillin, in clinical trials (Goldin et al. 1966; Roga et al. 1971). For example, neurologic symptoms, gastrointestinal toxicity or frequent skin rash have been previously described for free α-sarcin *in vivo* assays with mice (Goldin et al. 1966). Mitogillin has also been evaluated by repeated-dose toxicity tests performed in mice, dogs and monkeys (Roga et al. 1971). In the particular case of mice that received 0.2 mg/kg/day, only two of ten mice survived the test period, and all of them showed weight loss and organs injury (Roga et al. 1971). Similar effects were described in the other trials (Roga et al. 1971). This explains why in the 1970s these proteins were abandoned as promising antitumoral agents. However, none of these effects have appeared in the immunotoxin-treated mice, despite having used fivefold higher doses of immunotoxin (in nmoles) than those described for the ribotoxin. A result which again favours the interpretation, that the immunotoxin action is highly specific *in vivo*. Thus, IMTXA33αS shows both high efficacy and specificity against colon cancer tumors on *in vivo* assays with undetectable unwanted side-effects. Further steps in the future have to be considered including different via of administration, determination of the maximum tolerated dose of the molecule, limiting toxicities or a more thorough analysis of the effect on the different tissues and organs, among others. However, the results herein presented would be enough and consistent to be considered as a proof of concept.

As mentioned before, other immunoconjugates constructs have been previously used in preclinical or clinical trials based on mAbA33, most of them using the whole antibody molecule conjugated with radionuclides (Scott et al. 2005; Almqvist et al. 2006; Panjideh et al. 2008). In these clinical trials no toxicity in normal colon was observed, although GPA33 could be detected in the colonic epithelium (Scott et al. 2005; Almqvist et al. 2006; Ackerman et al. 2008; Panjideh et al. 2008). The surface persistence of the GPA33 antigen and normal intestinal epithelium shedding contribute to the very good retention in tumor tissue and the rapidly released form normal cells (Almqvist et al. 2006; Ackerman et al. 2008), supporting the selective localization into the tumor of mAbA33 immunoconjugates, as shown by in vivo biodistribution in mice models (Scott et al. 2005; Almqvist et al. 2006; Panjideh et al. 2008). Our results provide further evidence of the potential of GPA33 antigen as a specific tumor-marker for colon cancer becoming a good alternative to those actually used in therapy (Sliwkowski and Mellman 2013). In this sense, biodistribution studies with IMTXA33αS would provide useful information about half-life and localization of the immunotoxin and would be a step further in the thorough characterization of its antitumor action.

On the other hand, RNases from different origins have arisen as new candidates for the toxic domain of immunotoxins versus the commonly used ricin, *Diphtheria* or *Pseudomonas* toxins, to improve their efficacy or to circumvent immunogenicity or side adverse effects (Ardelt 2013; D’Avino et al. 2014). In regard to IMTXA33αS immunogenicity, neither ribotoxins as α-sarcin (Rathore and Batra 1996; Goyal and Batra 2000; Lacadena et al. 2007) nor the humanized scFVA33 domain used (Welt et al. 2003; Scott et al. 2005) would contribute to a potential immune response against the immunotoxin (Carreras-Sangrà et al. 2012), avoiding the inactivation of its antitumor action. This issue should be considered and confirmed when assayed in future clinical trials. In fact, in addition to the ribotoxins advantages mentioned above for their use as immunotoxins toxic moiety, the results show that nonspecific toxicity associated with ribotoxins is extremely reduced when included in an immunotoxin.

As mentioned in the Introduction, different ribotoxins have been used before as components of immunotoxins and assayed in vitro (Orlandi et al. 1988; Wawrzynczak et al. 1991; Better et al. 1992; Rathore et al. 1997; Goyal and Batra 2000). Unfortunately none of the ribotoxin-based immunotoxins was studied beyond a preliminary characterization, most probably due to their larger size, which could hinder their correct internalization into solid tumors, or to the low structural stability of the immunoconjugates prepared. IMTXA33αS is the first immunotoxin based on ribotoxins and the scFvA33 that has demonstrated its antitumor effectiveness on *in vivo* assays.

## Conclusion

IMTXA33αS, an immunotoxin based in ribotoxin α-sarcin, exhibits highly specific antitumor effectiveness in mice harbouring human colon tumor xenografts. It also exhibits anti-angiogenesis, anti-proliferative properties, and lacks adverse side effects. Thus, the results herein presented are a step further in the potential clinical application of IMTXA33αS and, in general, of ribotoxins-based immunotoxins.

## Materials and methods

### Materials

All chemicals were of molecular biology grade and used without further purification.

### Protein production, purification and in vitro characterization

IMTXA33αS was expressed in *Pichia pastoris.* Structural and *in vitro*-functional characterization of the isolated immunotoxin was performed as previously reported (Carreras-Sangrà et al. 2012; Tomé-Amat et al. 2012) before assayed *in vivo*. If needed, the purified immunotoxin was lyophilized and stored at −80°C until use.

### Cell line culture

Human colon carcinoma SW1222 and LIM1215 cells were provided by Dr. Carl Batt under the partnership Cornell University-Ludwig Institute of Cancer Research. SW122 cells were used as GPA33-positive cells for xenografts induction. Both cell lines were authenticated in 2010 by short tandem repeat (STR) analysis. Cells were also tested by flow cytometry, using a double labelling of GPA33 and CD44, characteristic of this cell line, immediately before any *in vitro* or *in vivo* assays. Double checking of GPA33 expression was also made by western blot using cellular extracts. Human colon adenocarcinoma HT-29 (ATCC:HTB-38) and epidermoid carcinoma A431 (ATCC:CRL-1555) cells were obtained from the ATCC Cell Biology Collection which carried out its characterization by STR analysis. Cells were grown as described (Carreras-Sangrà et al. 2012; Tomé-Amat et al. 2012) in Dulbecco’s modified Eagle’s medium (DMEM), containing glutamine (300 μg/ml), 50 U/ml of penicillin, and 50 μg/ml of streptomycin, and supplemented with 10% fetal bovine serum (FBS). Incubation was performed at 37°C in 5% CO_2_ humidified atmosphere. Harvesting and propagation of cultures were routinely performed by trypsinization. The number of cells used was determined using a haemocytometer.

### In vitro cytotoxicity assay

To evaluate the *in vitro* cytotoxic activity of α-sarcin-based constructions, inhibition of protein biosynthesis was determined as described (Lacadena et al. 2007; Carreras-Sangrà et al. 2012). GPA33-positive cells (SW1222 and LIM1215) and GPA33-negative cells (HT-29 and A431) were incubated with different concentrations of IMTXA33αS, free fungal wild-type α-sarcin or scFvA33. After 72 h of incubation at 37°C, the medium was removed and replaced with a fresh one supplemented with 1 mCi per well of L-[4,5-3H]-Leucine (166 Ci/mmol; Amersham, UK). After an additional incubation for 6 h, the medium was also removed and cells were fixed with 5.0% (w/v) trichloroacetic acid in PBS and washed three times with cold ethanol. The resulted dried pellet was dissolved in 0.2 ml of 0.1 M NaOH containing 0.1% SDS, and radioactivity was counted on a Beckman LS3801 liquid scintillation counter. To calculate the IC_50_ values (protein concentration inhibiting 50% protein synthesis) the results were expressed as a percentage of the radioactivity incorporated in control samples incubated without any of the three proteins, wild type α-sarcin, scFvA33 or the immunotoxin IMTXA33αS. Three independent replicate assays were conducted to calculate the average IC_50_ values.

### Animal treatment

All animal procedures were performed with the approval of the Complutense University Animal Experimentation Committee, according to the European official regulations.

Balb/c *nude* male mice (7 weeks old) were purchased from Harlan Laboratories S.A. (Barcelona, Spain) to evaluate the *in vivo* effect of IMTXA33αS against colorectal cancer induced xenografts. Two sets of assays were performed, using the Animal Facilities of either the Complutense University or the Centro Investigaciones Biológicas-Consejo Superior Investigaciones Biológicas (CIB-CSIC) in Madrid.

Mice were allocated into three experimental groups (n = 8): PBS (phosphate buffered saline), IMTX50 and IMTX100 (treatment with 50 or 100 μg of immunotoxin per injection, respectively). Prior to the experiments, animals were given a 7-day adaptation period with free access to food and water at all times. Each mouse received a subcutaneous injection into the right flank of 2×10^6^ SW1222 cells, resuspended in 100 μl PBS and 100 μl of Matrigel (BD Biosciences). Once the tumor volume raised 50–100 mm^3^, mice were injected intraperitonealy either with PBS or immunotoxin. Seven doses, every 48 hours, of PBS or the two different amounts of immunotoxin (50 or 100 μg) were given. Two control groups, free-α-sarcin wild type (n = 3) and scFvA33 (n = 3) were also included. In this case, we used doses of 40 and 61 μg per injection, respectively, which were equivalent to the highest dose of immunotoxin used (IMTX100), (2.2 nanomoles),

Tumors were routinely measured during this period with an external caliper, and volume was calculated as (width/2)^2^ x (length/2). Mice were also weighted throughout the experiment. At the end of the treatment, animals were sacrificed and tumors and organs were collected. Tumors were divided in portions and stored fixed in buffered 4% paraformaldehyde (PFA) or in Tissue-Tek (Sakura Finetek Europe) for immunofluorescence staining.

### ImmunofluorescenceTumor analysis

Histological analyses of excised tumors were performed. Tissue-teK embedded tumor sections of 8–10 μM thickness were obtained using a cryotome (Criocut Leica CM), fixed in methanol, washed with IFF buffer (5% FBS, 1% BSA, PBS) and incubated with anti-GPA33 polyclonal human mAb A33 (Santa Cruz Biotechnologies), anti-CD31 (Pharmingen/BD Biosciences), anti-Ki67 (Thermo Scientifics) or anti-Caspase3 (Cell Signaling Technology) antibodies. CD31, Ki67 and active-caspase3 are well-stablished markers for angiogenesis, proliferation and apoptosis, respectively. Secondary anti-rabbit antibodies, labelled with AlexaFluor 647 for GPA33 and Ki67 detection or anti-mouse AlexaFluor 488 for CD31 and Caspase3, were purchased from Invitrogen. Cell nuclei were stained with Prolong Gold with 40,6-diamidino-2-phenylindole (DAPI) (Molecular Probes). All these incubations were performed at room temperature. A Leica TCS P2 confocal microscope and the corresponding LCS lite software were used to obtain the fluorescence images. At least three sections of two representative tumors from each experimental group were used.

### Histopathological analysis

Brain, spleen, liver, kidneys, lungs and intestine were collected at the end of the treatment and fixed in 4% PFA. Collected organs were visually analysed for possible macroscopic changes resulting from treatment. Histological assessment of organs samples was performed by hematoxylin and eosin (H&E) staining. The samples were sliced into sections as described above, fixed in PFA and stained for H&E analysis. Morphological changes were evaluated using a light microscope and H&E-stained images were acquired.

### Statistical analysis

ANOVA with a post hoc analysis by the Student-Newman-Keuls’ test was used to compare variations in the mean tumor sizes at different treatment time points in each experimental group. Differences between experimental groups were considered statistically significant at P < 0.05. All values were expressed as arithmetic means ± sem (standard error of the media). For immunofluorescence tumor statistical analysis, at least three sections of two representative tumors from each experimental group were used.

## Abbreviations

ADEPT: Antibody-directed-enzyme-produg-therapy
DAPI: 40,6-diamidino-2-phenylindole
DMEM: Dulbecco’s modified Eagle’s medium
EGFR: Epidermal growth factor receptor
FBS: Fetal bovine serum
GPA33: Glycoprotein A33
GRAS: Generally regarded as safe
H&E: Hematoxylin and eosin
huA33: A33 humanized monoclonal antibody
JAM: Junction adhesion molecules
PBS: Phosphate buffered saline
PFA: Paraformaldehyde
RNases: Ribonucleases
scFv: Single-chain variable fragments
SRL: Sarcin/ricin loop
STR: Short tandem repeat
VEGF: Vascular endothelial growth factor
